# Recurrent horizontal transfer of arsenite methyltransferase genes facilitated adaptation of life to arsenic

**DOI:** 10.1038/s41598-017-08313-2

**Published:** 2017-08-10

**Authors:** Song-Can Chen, Guo-Xin Sun, Barry P. Rosen, Si-Yu Zhang, Ye Deng, Bo-Kai Zhu, Christopher Rensing, Yong-Guan Zhu

**Affiliations:** 1 State Key Lab of Urban and Regional Ecology, Research Center for Eco-Environmental Sciences, Chinese Academy of Sciences, Beijing, 100085 China; 2 Department of Cellular Biology and Pharmacology, Herbert Wertheim College of Medicine, Florida International University, Miami, Florida 33199 United States; 3 School of Civil and Environmental Engineering, Georgia Institute of Technology, 311 Forest Dr., Atlanta, GA 30332-0512 United States; 4 Key Lab of Urban Environment and Health, Institute of Urban Environment, Chinese Academy of Sciences, Xiamen, 361021 China; 5 University of Chinese Academy of Sciences, Beijing, 100049 China; 6 College of Agriculture and Life Science, Cornell University, Ithaca, 14853 United States

## Abstract

The toxic metalloid arsenic has been environmentally ubiquitous since life first arose nearly four billion years ago and presents a challenge for the survival of all living organisms. Its bioavailability has varied dramatically over the history of life on Earth. As life spread, biogeochemical and climate changes cyclically increased and decreased bioavailable arsenic. To elucidate the history of arsenic adaptation across the tree of life, we reconstructed the phylogeny of the *arsM* gene that encodes the As(III) S-adenosylmethionine (SAM) methyltransferase. Our results suggest that life successfully moved into arsenic-rich environments in the late Archean Eon and Proterozoic Eon, respectively, by the spread of *arsM* genes. The *arsM* genes of bacterial origin have been transferred to other kingdoms of life on at least six occasions, and the resulting domesticated *arsM* genes promoted adaptation to environmental arsenic. These results allow us to peer into the history of arsenic adaptation of life on our planet and imply that dissemination of genes encoding diverse adaptive functions to toxic chemicals permit adaptation to changes in concentrations of environmental toxins over evolutionary history.

## Introduction

Arsenic is a pervasive environmental toxin, and exposure to arsenic was no doubt one of the challenges to the origin of life^1^. Geologically, there have been many changes in global redox states and climates over the past 4.5 billion years^2, 3^, which dramatically influenced arsenic concentrations and bioavailability. Specifically, there were dramatic increases in marine arsenic concentrations in the late Archean eon (3–2.5 billon years ago (Bya)) and during interglacial periods after Huronian (~2.4–2.1 Bya) and Cryogenian glaciations (~0.85–0.58 Bya), respectively^4^. How organisms adapted to arsenic stress in response to the enormous shifts in biogeochemistry of arsenic may have influenced the evolution of modern life^1, 4, 5^. However, there is sparse evidence with respect to how life coped with toxic arsenic throughout geological time lines.

**Figure 1:**
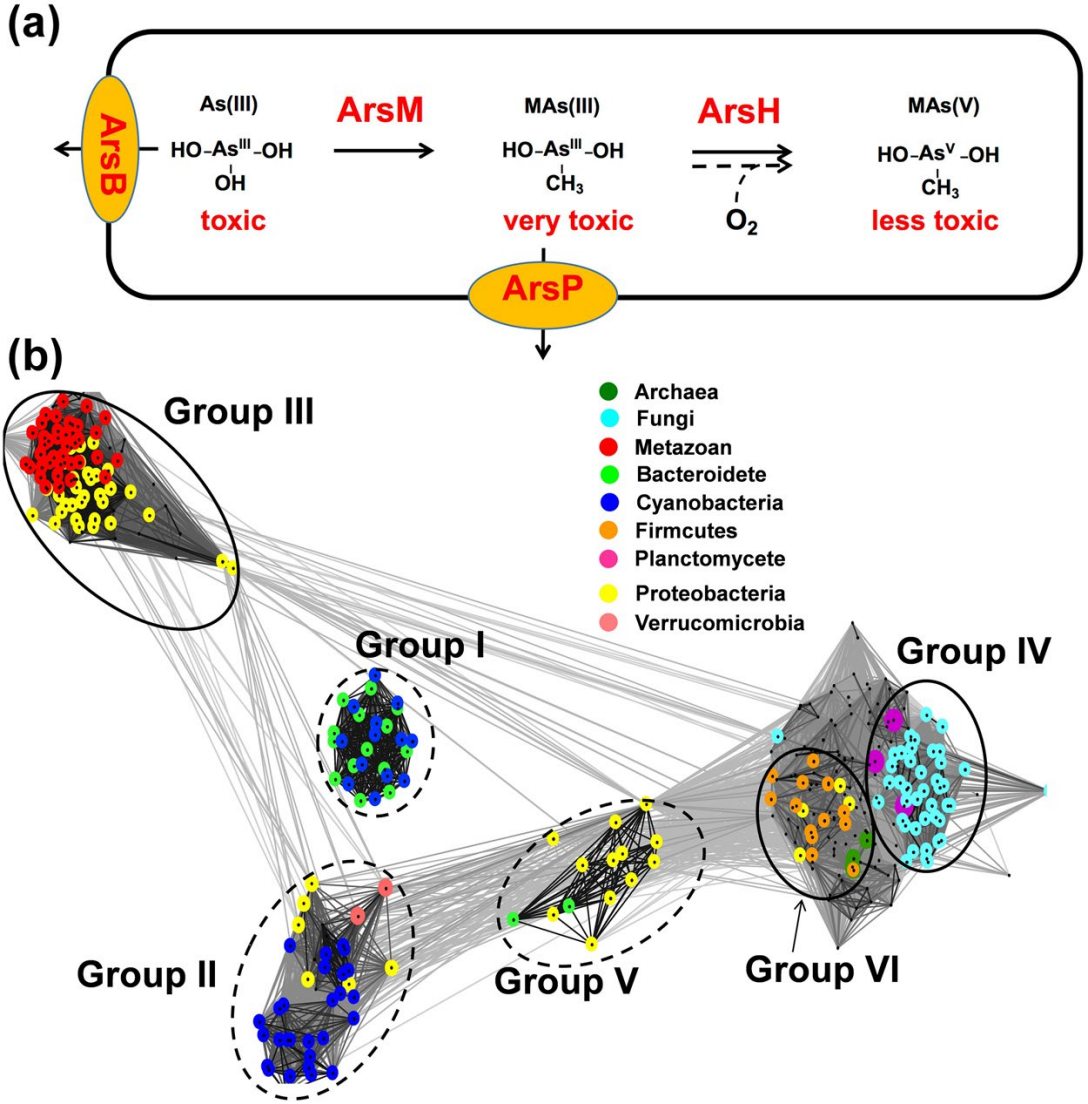
(**a**) Diagram representing enzymatic reactions catalyzed by arsenite efflux permease (ArsB), arsenite methyltransferase (ArsM), organoarsenical oxidase (ArsH) and methylarsenite efflux permease (ArsP). As(III): arsenite, MAs(III): methylarsenite, MAs(V): methylarsenate; (**b**) Cluster map of As(III) SAM methyltransferases (ArsM). Protein sequences of ArsM orthologs were clustered based on their pairwise similarity using the CLANS program (Frickey and Lupas, 2004). The clans containing sequences from more than one kingdom of life are indicated by black circles. Clans containing *arsM* from different phylums of bacteria are indicated by dash circles.

The composition of genomes and proteomes of extant organisms may bear imprints of ancient biogeochemical events^6–9^ that can help to reconstruct the evolutionary history of how life adapted to ancient changes in arsenic geochemistry. Multiple pathways for detoxification of both inorganic and organic arsenicals have been evolved in microorganisms. The genes for these defenses are usually encoded in arsenic resistance (*ars*) operons^10^. The widely distributed pathway for resistance is efflux of cellular arsenic from bacteria, archaea and fungi catalyzed by either the ArsB or Acr3 efflux permeases (Fig. 1a)^11^. A parallel pathway widely distributed across various kingdoms of life, for As(III) resistance, is methylation catalyzed by the As(III) *S*-adenosylmethyltransferase (ArsM, Fig. 1a), which converts As(III) into trivalent methylated species that are non-enzymatically oxidized to the non-toxic pentavalent species methylarsenate [MAs(V)], dimethylarsenate [DMAs(V)] and volatile trimethylarsine [TMAs(III)]^12, 13^. Metagenomic analysis indicates that the ArsM-catalyzed arsenic methylation strategy may be a major mechanism of resistance in extant marine cyanobacterial populations^14^. AS3MT, the animal version of ArsM, has been suggested to be the primary route of arsenic detoxification in mammals^15^. Although potentially significant from an evolutionary standpoint, the evolutionary history of ArsM, which encompasses imprints of how modern life adapts to arsenic stress and how the different kingdoms of life incorporated this machinery, has remained a mystery.

Horizontal gene transfer (HGT), the movement of genetic information among species, has been recognized as a significant mechanism for prokaryotic adaptation and diversification^16^. Recently, recognition of rapid expansion of newly sequenced eukaryotic genomes allows for the identification of additional examples of cross-kingdom HGT^17–19^, which contribute to the adaptive potential of the recipients. One report describes the discovery that an extremophilic eukaryote exploits horizontally-acquired genes for environmental adaptation from other kingdoms, e.g., bacteria and archaea^20^. The widespread occurrence of *arsM* genes among various kingdoms of life raised the question whether cross-kingdom HGT played a role throughout the evolutionary history of the ArsM As(III) SAM methyltransferase.

To understand the natural history of arsenic adaptation, we analyzed more than 3600 annotated genomes for putative *arsM* genes. The phylogenetic distribution, geological timing and evolutionary history for *arsM* were determined using phylogenomic methods. By examining these data, we infer the timing and complex genetic events that have marked the history of *arsM*, representing the genomic fossils that illustrate how life adapts to toxic arsenic throughout evolutionary history.

## Results

### Distinct phylogenetic groups

In this study the sequences of 231 bacterial, 19 archaeal and 93 eukaryotic ArsM orthologs were identified in the eggNOG database. Among all the retrieved ArsM sequences, cysteine residues integral to the binding of arsenite to ArsM protein at positions 72, 174 and 224 in CmArsM^21^ are conserved. In addition, SAM-binding motifs are also conserved among all ArsM sequences. The similarity in these key catalytic residues and domain structure indicate the homology of all ArsMs. The phylogenetic distribution of *arsM* genes is extensive, including bacteria and archaea, animals and fungi, illustrating the widespread occurrence of the potential for methylation of inorganic arsenic^22^. Cluster mapping of ArsMs indicates that they segregated into at least 6 distinct groups (Fig. 1b), each of which is comprised of sequences from quite different organisms. Group I consist of ArsMs from cyanobacteria and bacteroidetes phyla. Group II are found in members of cyanobacteria, firmicutes, verrucomicrobia and proteobacteria. Group III are predominantly composed of members of the metazoan, protist, red algae and proteobacteria. Group IV are from fungi and planctomycetes. Group V composed of ArsMs from anaerobes, including chlorobia and anaerobic proteobacteria. Group VI contains ArsMs from archaea and various phyla of bacteria. The observation that sequences from remotely related organisms are closely related, and that they cluster together in a way that is vastly different from the species phylogeny, suggested that evolution of ArsM might not be vertical.

**Figure 2:**
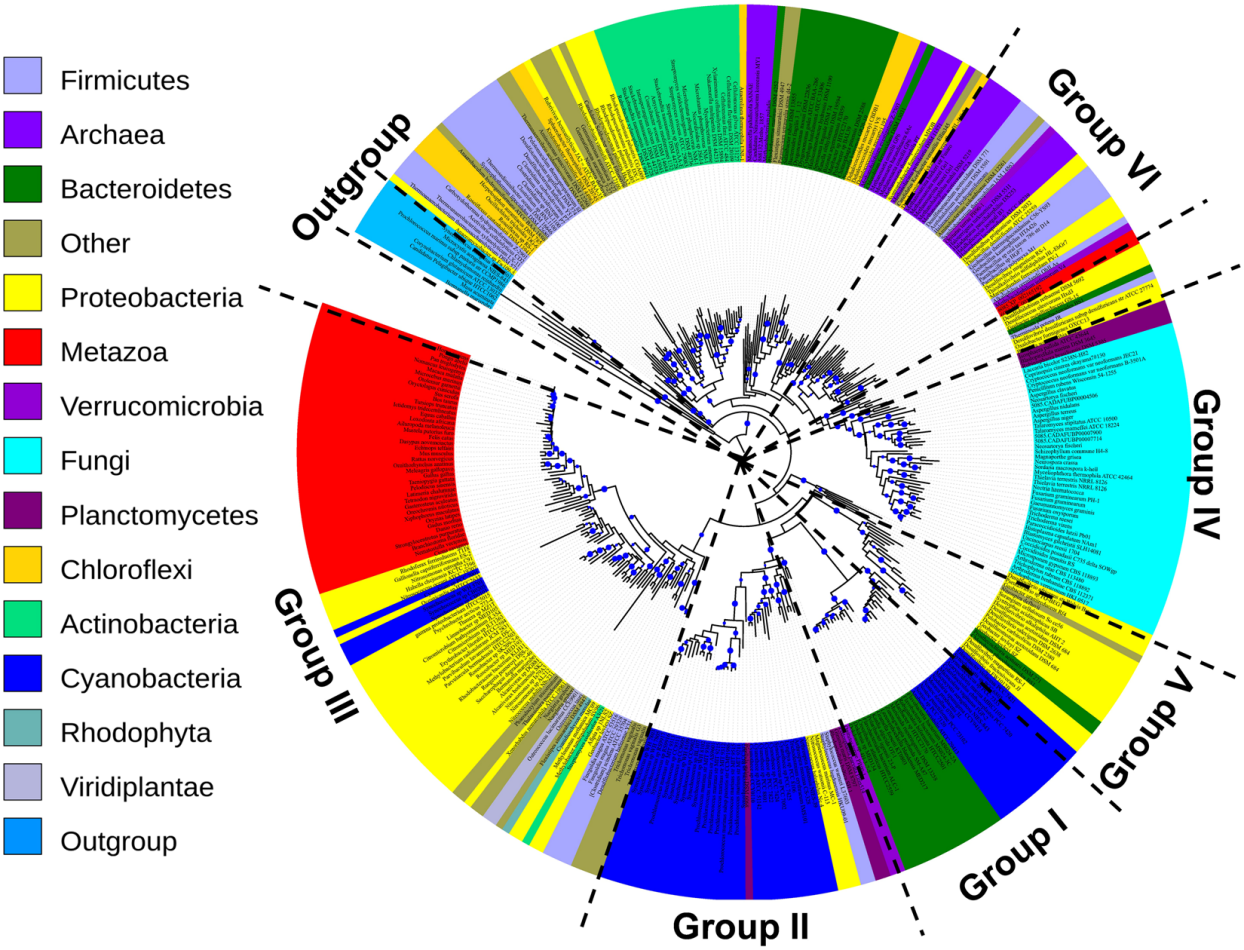
Phylogeny of ArsM As(III) SAM methyltransferases. The phylogenetic tree was constructed using the maximum likelihood program RAxML. The statistical significance of the branch pattern was estimated by conducting 100 bootstrap replications of the original amino acid alignment; bootstraps >50 were shown as blue circle. The detailed trees of each group are provided in Fig. 3, Figure 4–6 The related SAM-dependent mycolic acid cyclopropane synthetase (*cmaS*) genes were used as outgroup^14^.

To further explore the evolutionary history of ArsM, phylogenetic analysis was performed for all ArsM homologs. Consistent with the results of cluster mapping, an extraordinary incongruence between our ArsM tree (Fig. 2) and the published phylogeny^23^ of eukaryotic species was detected, e.g., ArsM homologs in eukaryotes showed a non-monophyletic distribution in three distinct groups with well-bootstrapped support (Group III, IV and VI, Fig. 2). By examining the entire Bayesian posterior ArsM tree samples, we found that none of the trees resolved ArsM from the eukaryotes as being monophyletic. In addition, AU test implemented in CONSEL package rejected (P < 0.01) the alternative tree with monophyletic eukaryotic sequences (Table S1). Here we investigate and discuss the possible causes of the conflict pattern between ArsM tree and species tree.

Bacterial contaminations during genome sequencing of eukaryotes are not likely to be the explanation for the clustering of eukaryotic and prokaryotic ArsM (Figs 1 and 2), because ArsM homologs in eukaryotes, e.g., fungi^24^ and animals^25^, have been well characterized. All eukaryotic *arsM* genes except that in *Hydra magnipapillata* carry introns (Table S2). Although *arsM* genes in *H. magnipapillata* were found lack introns, they are closely linked to eukaryotic genes with introns on the same genomic scaffolds (Table S2), suggesting that these sequences do not represent bacterial artifacts.

Next, we investigated whether aberrant evolutionary substitution process may have misled the phylogenetic reconstruction. For example, heterogeneous evolutionary rate across lineages can obscure true phylogenetic signal^26^, thereby causing a gene tree to be incongruent with the species tree. To test whether eukaryotic lineages in three different groups (Group III, IV and VI) evolved at different evolutionary rates, we applied a likelihood ratio test (LRT) between a model in which all eukaryotic lineages evolve at the same rate (homogeneous model) and a model in which different lineage rates are allowed (heterogeneous model). An LRT showed that the homogeneous model cannot be rejected (Table S3), which indicated the difference in evolutionary rates among the eukaryotic lineages is small. Similarly, relation rate test did not show significant difference among evolutionary rates of these eukaryotic lineages (Table S3). Taken together, the evolutionary rate of ArsM among different eukaryotic lineages appear to be similar and are not likely to explain the incongruence between the gene tree and species tree.

Differential loss was suggested to be one of the major reasons for the patchy distributions of genes across eukaryotic lineages^27^. If this has been the case, all eukaryotic ArsMs should be expected to cluster in a monophyletic group with certain taxonomic lineages missing. However, the converse was observed: ArsMs distributed in non-monophyletic clusters with prokaryotes in ArsM tree (Fig. 2). We therefore hypothesized that the incongruent ArsM tree could be the result of multiple HGT events among prokaryote and/or eukaryote.

### Early origin of As(III) methylation

**Figure 3:**
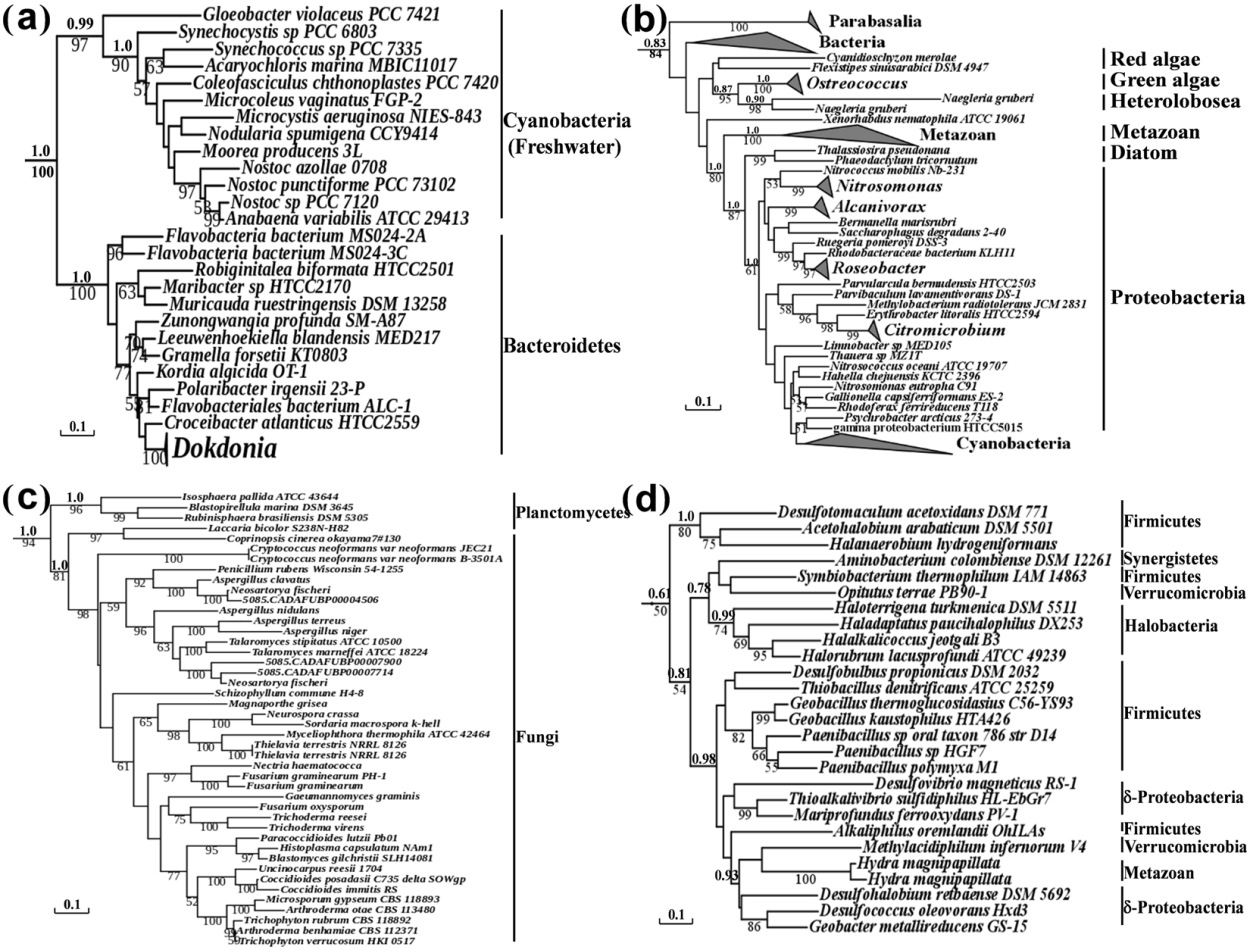
Maximum likelihood tree of ArsM orthologs in different groups. (**a**) Maximum likelihood tree of ArsM orthologs in Group I; (**b**) Maximum likelihood tree of ArsM orthologs in Group III; (**c**) Maximum likelihood tree of ArsM orthologs in Group IV; (**d**) Maximum likelihood tree of ArsM orthologs in Group VI. Maximum likelihood bootstrap support value (>50) are indicated below branches. Bayesian posterior probability values of critical bipartition appear above the branch for comparison.

ArsM sequences of cyanobacteria in Group I (Fig. 3a) form a strongly supported monophyletic clade (ML/Bayes = 97/0.99), and its phylogeny is generally congruent with 16 S rRNA phylogeny^28^. Therefore, the phylogeny of cyanobacterial ArsM in Group I corresponds to that of parent species, indicating linear evolution without HGT. The similarity of topology extends to the details that *Gloeobacter* is the earliest branch within ArsM phylogeny^29^, which is in line with the 16S rRNA tree (Fig. S1). This result indicates that the Group I-like ArsM was present before the divergence of *Gloeobacter* from other cyanobacteria. The evolutionary history of Group I version of ArsM appears to be dated back to an era before the last cyanobacterial common ancestor (LCCA). According to the fossil record and molecular dating, the LCCA is believed to have existed as early as 3 Bya^30^, considerably before the accumulation of oxygen in the atmosphere (2.45–2.32 Bya) during the Great Oxidation Event (GOE)^31^. Overall, the finding of an *arsM* gene in LCCA implies that ArsM arose in an anoxic world before the GOE. This result calls the primordial role of ArsM as a detoxification pathway into question: methylation of arsenite increases toxicity by producing highly toxic MAs(III) and DMAs(III), which are thermodynamically stable in the oxygen-free environment that existed before the GOE^32^. Thus, ArsM would not be a detoxification enzyme unless there were oxygen to convert the products to less toxic MAs(V) and DMAs(V). We postulate that the enzyme originally evolved to produce extremely toxic trivalent methylarsenicals that functioned as antibiotics to kill competitors in the primordial environment; after the GOE the trivalent species were nonenzymatically oxidized to pentavalent forms, transforming ArsM into a detoxifying enzyme^33^. Under this model, the original antibiotic producer (ArsM hosts) protected itself by pumping out its own toxic products (MAs(III)) with the methylarsenite efflux permease (ArsP)^34^, which simultaneously kill off its neighbors. This hypothesis is exemplified by the observation that *arsP* co-exists with *arsM*, primarily in anaerobes (Fig. S2b). In addition, clustering of *arsP* in anaerobic hosts in a monophyletic clade (Group V) in ArsM phylogeny (Fig. S2a) suggests that clustering of ArsP and ArsM might be an ancestral feature in lineages of anaerobes, supporting our hypothesis that ArsP evolved for detoxification of MAs(III) in ArsM hosts.

Alternatively, ArsM could function as arsenite detoxification pathway in the anoxic environment of the primordial earth through co-evolution with ArsH, which enzymatically oxidizes of MAs(III) to MAs(V)^35^. However, biochemical studies and cluster analysis suggests that this explanation is unlikely. First, ArsH uses molecular oxygen in its catalytic mechanism^35^, so it most likely evolved after the appearance of atmospheric O_2_ rather than functioning in the primordial anoxic environment. Second, ArsH and ArsM show a distinct taxonomic distribution, which suggests an independent evolutionary trajectory rather than co-evolution (Fig. S3b). Third, cluster analysis shows that ArsH and ArsM coexist primarily in aerobes, not anaerobes (Fig. S3c). This implies that it is organisms with an aerobic lifestyle that possess both ArsM and ArsH and suggests that ArsH did not evolve to play a role in detoxification of MAs(III) in ArsM hosts in anoxic environments.

### HGT of *arsM* from bacteria to metazoa

HGT of *arsM* genes may have contributed to the evolution of metazoans by allowing them to adapt to ubiquitous presence of arsenic. The metazoan group forms a strongly supported (ML/Bayes = 80/1.0) sister group to the bacteria/diatom group (Fig. 3b; Fig. S4). Formation of this cluster indicates that the metazoan group originated via the first ancient HGT from bacteria to the ancestor of the metazoan lineages. All metazoan ArsMs, including those from early-branching metazoan lineages (cnidaria), form a well-supported monophyletic clade (ML/Bayes = 100/1.0), so it is likely that the bacterial *arsM* gene was transferred to the common ancestors of all metazoans (urmetazoan) around 600–700 million years ago (Mya)^36–38^, and subsequent later gene loss might explain for differential presence of *arsM* gene among metazoan genomes. Urmetazoans possibly acquired *arsM* from bacteria that formed their food source through the operation of a gene transfer ratchet mechanism^39, 40^. In addition, a second but more recent HGT from bacteria to metazoan may also have occurred. As shown in Fig. 3d, *Hydra magnipapillata* are nested within bacterial species in ArsM phylogeny, which indicates a recent HGT from bacteria to this member of the Cnidaria phylum. The acquired *arsM* may have alleviated arsenic stress by biotransformation of arsenic into the less toxic species such as those commonly found in mammalian urine^40^. These observations suggest that HGT had a critical role in the adaptation of metazoans to environmental arsenic stress.

### HGT of *arsM* from bacteria to diatoms

We propose that the acquisition of an *arsM* gene by eukaryotes from bacteria conferred diatoms with the ability to cope with stressful marine arsenic concentrations. ArsM from two diatoms, *Thalassiosira pseudonana* and *Phaeodactylum tricornutum*, form a sister group to a bacterial-only clade with high bootstrap support (ML/Bayes = 87/100, Fig. 3b), indicating that an *arsM* gene was transferred from bacteria to diatoms. Although HGT is considered rare, with limited evolutionary significance in eukaryotes compared to prokaryotes, our results are supported by the observation that HGT between bacteria and diatoms is pervasive and occurs more frequently than that in other eukaryotes^41^. The occurrence of bacterial *arsM* genes in diatoms serves as part of a generally metabolic strategy in addition to the role in arsenic detoxification. For example, ArsM not only permits diatoms to produce less toxic MAs(V) or DMAs(V) but also provides the substrates for biosynthesis of organoarsenical compounds such as arsenosugars and arsenolipids^42, 43^. Arsenolipids, for example, are reported to compensate for the deficiency of phosphorus in diatoms growing in phosphorus-depleted oceans^44^. Arsenolipids have a phosphate sparing effect in synthesis of membrane phospholipids^45^.

### HGT of *arsM* from bacteria to fungi

Phylogenetic analysis of Group IV indicates a planctomycetes origin for fungal ArsM orthologs (Fig. 3c). ArsM orthologs from fungi form a well-supported clade (ML/Bayes = 94/1.0), and cluster as a sister group to the planctomycetes clade (Fig. 3c), suggesting that an ancestral fungal species acquired an *arsM* gene from a planctomycetes. The species-specific *arsM* gene duplications in *Aspergillus fumigatus* and *Thielavia terrestris*, respectively (Fig. 3c), indicate *arsM* genes evolve dynamically by gene duplications after ancestral acquisition. Although fungi represent the most recalcitrant of all organisms to gene transfer due to their robust cell walls and lost phagotrophic capacities, conjugation-like transfer and ecological association with other microorganisms could have promoted HGT between prokaryotes and fungi^46^. A phylogenomic analysis of 60 sequenced fungal genomes suggests that the gene encoding ArsC, a bacterial arsenite reductase which catalyzes a critical step in arsenate metabolism^1^, was transferred into lineages of the fungal kingdom^47^. In addition, the fungal species *A. fumigatus* has more arsenic resistance genes than any other eukaryote, and they all resemble bacterial arsenic resistance determinants. The original HGT must have been at least one entire bacterial *ars* operon. This includes ArsH, which gives MAs(III) resistance by oxidation to MAs(V)^35^, implying that *A. fumigatus* comes into contact with both inorganic and organic arsenicals. The acquisition of bacterial arsenate reductase genes as well as other arsenic resistance genes by fungi reflects the arsenic dilemma faced by fungi during their evolution trajectory. Thus, HGT of multiple *ars* genes from bacteria to fungi, including those involved in As(V) reduction, As(III) methylation and MAs(III) oxidation, provides synergistic effect in arsenic tolerance.

### HGT of *arsM* from bacteria to archaea

HGT of *arsM* also occurred between bacteria and archaea. The archaeal ArsMs are paraphyletic clustered or nested within other bacterial sequences (Fig. 2), indicative of several independent bacteria-to-archaea HGT events. For example, ArsM sequences from 4 halobacteria are nested within firmicutes and proteobacteria (Fig. 3d), indicating that the ArsM of the halobacterium group originated via HGT from bacteria, although the identification of the donor lineage is not straightforward. As(III) methylation represents a useful ecological function for the halobacteria and other extremophilic archaea. The natural habitat of halobacteria is often rich in heavy metals and metalloids, including arsenic^48^. In addition, other archaeal species are extremophiles, living in extreme environments such as hot springs, where the arsenic concentration is often very high^1^. Therefore, in addition to utilizing arsenic for bioenergetics purpose^49, 50^, acquiring an arsenite methylation pathway might be critical for adaptation of archaea to extreme environments^51, 52^.

### Eukaryote-to-eukaryote HGT of *arsM*

A eukaryote-to-eukaryote HGT of *arsM* gene may also have occurred between heterolobosea and green algae. The grouping (ML/Bayes = 95/0.87) of *Naegleria gruberi* (heterolobosea) and green alga in the ArsM phylogeny is surprising (Fig. 3b), as *Naegleria* and green alga are classified into different supergroups of eukaryotes^23^. A plausible explanation could be that the *arsM* gene from a green alga was transferred into *N. gruberi* through a secondary endosymbiotic event, as suggested by the ‘plastid-early’ hypothesis^53^. According to this evolutionary scenario, a secondary endosymbiosis of green alga occurred in the common ancestor of heterolobosea and euglenida, and the plastid-lack heterolobosea (*N. gruberi*) secondarily lost its plastid, after successful integration of an *arsM* gene into its nuclear genome. The endosymbiotically-transferred *arsM* gene logically would have allowed *N. gruberi* to adapt to high arsenic concentrations in its ecological niche. For example, the capacity for As(III) methylation provides *Euglena gracilis*, a protist related to *N. gruberi*, with a distinct arsenite metabolism that allows it to cope with arsenic stress in an acid drainage environment^54^. In addition, fusion of putative *arsM* genes and selenophosphate synthetase (SelD), as observed in the genome of *N. gruberi*, is advantageous to a free-living protist faced with potentially threatening environments^55, 56^.

### HGT of *arsM* between cyanobacteria and other bacteria phyla

Cyanobacteria do not form a monophyletic clade in the ArsM tree. One cyanobacterial group (Group II, Fig. S5), including *Synechococcus*, *Prochlorococcus*, *Cyanothece*, *Lyngbya*, *Trichodesmium erythraeum* and *Arthrospira*, clusters within firmicutes, proteobacteria and verrucomicrobia with high bootstrap support (ML/Bayes = 100/1.0). The remaining cyanobacteria (Group I, Fig. 3a) form a well-supported monophyletic group (ML/Bayes = 97/0.99). The paraphyletic grouping of cyanobacterial ArsM strikingly conflicts with 16 S rRNA-based phylogenies, which tends to place cyanobacteria as one coherent (monophyletic) phyla in bacteria^57^, indicating HGT occurred between cyanobacteria and other bacterial phyla. The current cyanobacterial ArsM topology is most easily explained by displacement of the original *arsM* gene in Group II cyanobacteria with an inter-phylum transferred ortholog from other Group II bacteria (xenologous gene displacement). Inter-phylum transfers between cyanobacteria and other phyla should not be surprising, as it has been estimated that individual cyanobacterial genomes acquired between 9.5% and 16.6% of their genes through HGT^58^. HGT between Group II organisms is further corroborated by the ecology of these species: Group II organisms are frequently found in sea bacterioplankton communities, providing a viable environment for gene sharing^59^. The alternative hypothesis - that both Group I and Group II-like *arsM* genes were present in the last common cyanobacterial ancestor and subsequently differentially lost - is less likely for several reasons^60^. First, all extant cyanobacteria have either Group I or Group II versions of ArsM - none has been found to encode both - which argues against retention of both versions in a single genome over a long evolutionary timescale, as required by an “ancient paralogy and differential loss” scenario^59^. Second, too many parallel independent losses would have to be postulated if the both Group I and Group II-like ArsMs were ancestral to cyanobacteria, which is not parsimonious^18^.

ArsMs from marine cyanobacteria (Group II, Fig. S5) and those from freshwater cyanobacteria (Group I, Fig. 3a) form phylogenetically-distinct clusters, suggesting that a HGT that occurred in marine cyanobacteria contributed to niche differentiation of these two cyanobacteria “ecotypes”. Marine cyanobacteria are more likely to suffer arsenic stress than those in freshwater due to higher arsenic concentrations in open seawater^61^. In addition, because arsenate is taken up by phosphate transport systems, low phosphorus concentrations in the ocean may contribute to higher levels of arsenate uptake, thereby increasing the stress of arsenic to marine organisms^62^. Consistently, a number of marine cyanobacteria are more efficient in arsenic methylation than freshwater cyanobacteria^63^. Natural selection has favored human AS3MT haplotypes that associate with more efficient arsenic metabolism in populations with generations of severe arsenic exposure as a result of increased expression of the gene^64^, and we suggest that HGT followed by selection for higher *arsM* expression in marine cyanobacteria that resulted in an increase in arsenic methylation rates may contribute to the ability of cyanobacteria to thrive in arsenic-rich marine environments.

### HGT of *arsM* among anaerobes

Inter-phylum HGT of ArsM between distantly related anaerobic bacteria may have played a role in their adaptation to an ecological niche. As shown in Fig. S6, ArsMs from two strictly anaerobic chlorobi, *Prosthecochloris aestuarii* and *Chlorobium phaeobacterioides*, are nested within a branch of strictly anaerobic genera of δ-proteobacteria, such as sulfur-reducing bacteria and ferric-iron reducing bacteria, strongly suggesting a transfer event from δ-proteobacteria to chlorobi. Although genetic exchange between distantly related organisms representing different bacterial phyla is thought to be very infrequent, extensive HGT within anaerobic mesophilic environments was previously described between *Sphaerochaeta* spp and *Clostridia* ^65^. Indeed, there is a higher frequency of HGT within anaerobic rather than aerobic bacteria, and a greater niche overlap and/or physical proximity among organisms within anaerobic environments favors HGT^66^. Inter-phylum HGTs appear to be fundamental for the adaptation of the anaerobes to their perspective ecological niches, although the ecological role of ArsM in anoxic environments is still unknown. These results suggest that the capabilities for As(III) methylation under anaerobic conditions were transferred between distantly related phyla.

## Discussion

The concentrations and bioavailability of arsenic varied dramatically during major periods of phylogenetic diversification. As a result, there was early evolution of an arsenic methylation gene that then continually transferred horizontally among kingdoms of life. These HGT events mark the natural history of arsenic adaptation. Our phylogenetic analysis suggests an early cyanobacterial ArsM origin toward the end of the Archean Eon, more than 2.5 Bya (Fig. 3a). In the geological records, the concentration of arsenic in the late Archean Eon (3 Bya-2.5 Bya) increased in oceans^67^, probably due to significant oxidative weathering of arsenic-rich minerals triggered by low levels of O_2_ available before the GOE^68–71^. Hence, the innovation of arsenic methylation systems in cyanobacteria at the end of Archean could have produced fitness advantages, leading to an increase in cyanobacterial diversity and abundance^57^, which in turn would supercharge cyanobacteria, the engine of life. Subsequently inter-kingdom HGT of *arsM* represented a major mechanism of evolutionary adaptation to arsenic stress. Our results suggested that the arsenic methylation pathway found in the common ancestor of metazoa (~600 Mya) was acquired via HGT (Fig. 3b; Fig. S4), quite likely as a result of an ‘arsenic burst’ after the Cryogenian glaciations (850–580 Mya)^4^. In this scenario, a burst of deglaciating metal/As-rich waters and intensified chemical weathering^4^ would have led to massive increases in the concentration and bioavailability of arsenic in the ocean, selecting for stable transfer of *arsM* from prokaryote to metazoa. We proposed that increased fitness of ArsM-containing species led to a higher frequency and wider distribution of metazoa during post-Cryogenian.

**Figure 4:**
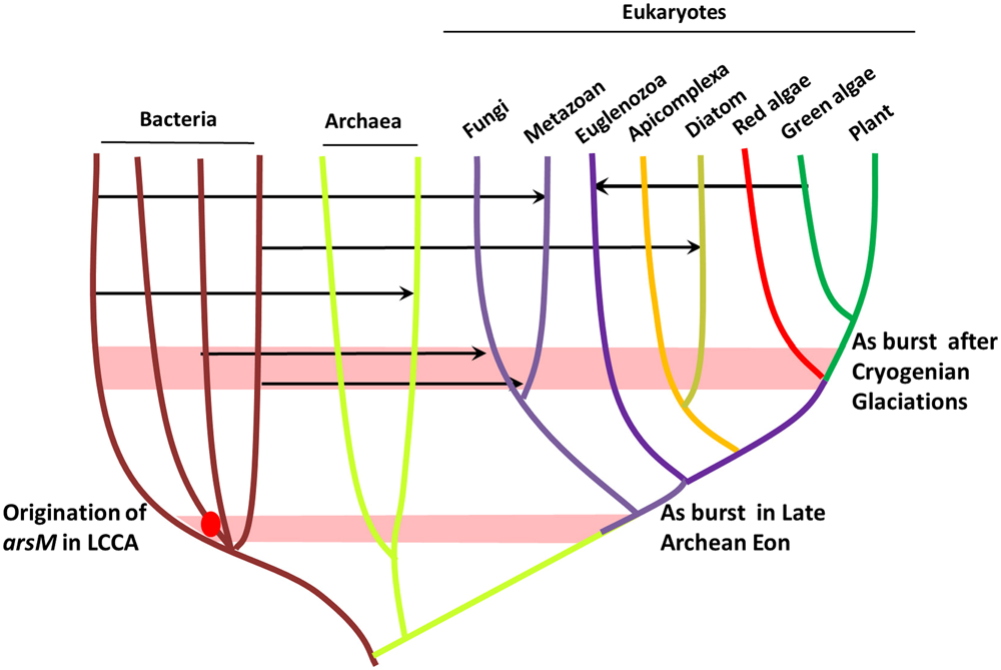
Diagram illustrating the inter-kingdom HGT of *arsM* gene. Horizontal lines and arrows show HGT donors and recipients. LCCA, last cyanobacterial common ancestor. Information about tree of life is based on Yue *et al*.^17^.

Given that ArsM may have played a critical role in promoting the adaptation and diversification of life under arsenic changing environment, our study has implications for the evolutionary significance of inter-kingdom HGT. Conventional belief is that HGT between distantly related organisms from different kingdoms is infrequent, especially when multiple cellular eukaryotes are involved. However, our analysis reveals that at least 6 HGT events (in the case of *arsM*) occurred among different kingdoms of life (Fig. 4). An extremophilic eukaryote was recently suggested to exploit horizontally-acquired genes in adaptation to toxic metal-rich environments from other kingdoms, e.g., bacteria and archaea^20^. Therefore, inter-kingdom HGT has been overlooked as a potentially significant factor in facilitating adaptation of organisms to changing environments and/or the development of resistance to not only arsenic but also other environmental toxic substances^72–74^.

Two lines of evidence further suggest that the horizontally transferred *arsM* genes provide an adaptive function to their hosts. Firstly, signatures of purifying selection were observed acting on the *arsM* genes from HGT hosts, such as metazoa, fungi, diatoms and archaea, as indicated by the overall gene dN/dS ratio (less than 1) of these gene groups (Table S1). Consistently, individual codons with significant (*P* < 0.05) signatures of purifying selection were identified in different *arsM* groups (Table S1), and no sites were found to be under positive selection. These results imply that *arsM* genes were under rigorous functional conservation in response to strong selective pressure after HGT, which highlights the significant role of *arsM* in allowing their host to adapt to novel ecological niches. Secondly, the majority of ArsM proteins from HGT hosts possess four cysteine residues integral to arsenite binding and flanking motifs of their corresponding bacterial ArsM groups (Fig. S7), indicating the retention of enzymatic function after HGT. The domestication of ArsM function within recipient organisms is further demonstrated by a genome-wide association study among human populations living in a region with elevated arsenic exposure shows that adaptive polymorphisms in the AS3MT gene, the metazoan version of *arsM*, play a role in human acclimatization to arsenic rich environments^64^.

An emerging pattern is that most inter-kingdom HGT of the *arsM* gene involve acquisition from bacteria. For example, eukaryotic ArsM, including those in metazoa, fungi, diatoms, can be traced back to multiple acquisitions from independent bacterial groups (Fig. 4). Although we also propose eukaryote-to-eukaryote HGT (Fig. 3b and 5), current evidence indicates that bacteria appear to be the common candidate donors of new physiological functions for eukaryotes^20^. Indeed, bacteria-to-eukaryote HGT underlies the adaptation of many microbial eukaryotes^20, 59^. Bacteria are abundant in nearly every ecological niche, and many eukaryotes survive by eating and digesting them in large numbers^75^, so bacterial genes, including *arsM*, are a source of foreign genetic material for eukaryotes. Secondly, bacteria are metabolically more diverse than eukaryotes, and, as such, have more to offer in the way of genetic information for new and beneficial functions such as arsenic methylation. In addition, many bacteria have mechanisms such as the type IV secretion system to transfer DNA to other organisms^76^. These ecological links between donor and recipient lineages further imply that bacteria provide a rich source of genetic material that allows rapid adaptations to new ecological niches, in this case, arsenic stress.

The finding of recurrent HGT events during the evolution of ArsM raises the question of why ArsM was so frequently transferred horizontally among different kingdoms of life. Genes are logical candidates for domestication after HGT if 1) they independently provide functionality and 2) are immediately beneficial to a recipient organism^18^. *arsM* genes, which were transferred from various bacteria to other kingdoms of life, meet these qualifications. Firstly, ArsMs contain both SAM binding and catalytic domains, and require no other proteins for their activity^21^; secondly, the ability to methylate arsenic into innocuous organic forms is of immediate advantages for organisms suffering from environmental arsenic stress. As such, ArsM could easily be domesticated as a single functional unit in other kingdoms of life such as metazoans, diatoms, archaea and fungi (Fig. 4). In the case of recurrent transfer and retention of a gene encoding a specific protein in diverse species, a model that allows for immediate selection seems more likely^18^, although many genes transferred between prokaryotes are pre-adaptive traits with neutral value^77^.

In summary, we demonstrate here that life largely avoided arsenic stress in the late Archean Eon and Proterozoic Eon, respectively, by the propagation of *arsM* genes and bacterial genes encoding As(III) S-adenosylmethyltransferase were horizontally transferred in at least six distinct events to diverse kingdoms of life. The recurrent and independent transfer of *arsM* genes to distinct kingdoms of life suggests that these genes confer immediate fitness benefits by supplying a new dominant function to the host organisms. Diverse genetic and biochemical mechanisms have been demonstrated to allow organisms to physiologically adapt to the stress of toxic environmental substances^78^. We propose that the spread of genes that underpinning the ability to tolerate toxic substances - beyond the *arsM* genes – has occurred multiple times among different kingdoms in response to selective pressures imposed by changing environmental conditions through geological history.

## Methods

### Identification of arsM genes and cluster mapping

To obtain sequences of arsM genes encoding candidate orthologs, the Clusters of Orthologous Groups (COG) entry associate with ArsM function, NOG07857, was used to extract additional ArsM sequences in the eggNOG^79^ database. Conserved domains of the extracted protein sequences were identified with the Batch Web CD-Search Tool in the NCBI Conserved Domain Database (CDD) (http://www.ncbi.nlm.nih.gov/Structure/bwrpsb/bwrpsb.cgi). To identify the As(III) binding site of ArsMs, each extracted protein sequence was aligned to the reference alignment^80^ of known ArsM sequences with hmmalign program from the HMMER3 package (http://hmmer.janelia.org). Only those sequences containing SAM-binding domains, as well as at least 3 of the four conserved cysteine residues corresponding to residues 44, 72, 174 and 224 in the ArsM sequence of the eukaryotic red algae *Cyanidioschyzon merolae* sp. 5508 (CmArsM)^21^, were used for further analysis. Sequences of the product of the cmaS gene, which encodes the closely related SAM-dependent mycolic acid cyclopropane synthetase were also included as an outgroup, as previously described^14^. All arsM sequences were compared with each other using BLASTP and clustered using CLANS software^81^. The final visualization was obtained with an e-value threshold of 1e-40 applied in the CLANS program.

### Identification of ArsP and ArsH

To identify ArsP sequences in eggNOG database, ArsP orthologs in bacteria (ENOG4105DSX) and Archaea (arCOG02712) were retrieved (no orthologs was found in eukaryotes). Only sequences with the conserved “PXCSCXXXP” motif were considered to be ArsP orthologs. To obtain putative ArsH orthologs, an initial phylogenetic tree of COGs associate with ArsH function from eukaryotes (ENOG410IITQ), bacteria (ENOG4105CAU) and archaea (arCOG04624) in the eggNOG database was built (see phylogenetic reconstruction). The sequences in the clade which includes known ArsH function was retrieved as putative ArsH orthologs (Fig. S3a). Information about oxygen requirement associated with these organisms was acquired from either NCBI or Genomes OnLine Database (GOLD).

### Phylogenetic reconstruction

The selected protein sequences were aligned using several algorithms, including MUSCLE, CLUSTALX, MAFFT-L-INS-i and T-coffee. The MUSCLE aligned sequences, which yielded the highest score according to the word-oriented objective function (WOOF), were chosen for subsequent analysis^82^. Low quality alignment regions were removed by TrimAl to retain only those columns present in at least 30% of the sequences^83^. Alignments were visually inspected and manually edited when necessary. ProtTest was used to find the most suitable model for phylogeny reconstruction before phylogenetic analysis^84^. The maximum-likelihood (ML) phylogenetic tree was constructed using RAxML version 8.2.0^85^ with the WAG Model (+I + G), which got the highest score in the ProtTest analysis. Support values were calculated using 100 bootstrap replicates. A Bayesian tree was constructed using MrBayes version 3.2.6 with the WAG model^86^. For the MrBayes consensus trees, 5,000,000 generations were completed with trees every 500 generations. To investigate the extent of selection affecting the arsM genes, SLAC (Single Likelihood Ancestor Counting) in the HyPhy software suite^87^ were used to perform the calculation of non-synonymous and synonymous (dN/dS) ratio, as well as statistical tests for purifying or positive selection for individual codons. Additional statistical tests in the HyPhy software suite REL (Random Effect Likelihood) and FUBAR (Fast Unbiased Bayesien Approximation) were used to confirm whether arsM codons display statistically significant signature of purifying selection.

### Phylogenetic congruence Test

To determine whether the eukaryotic non-monophyly in ArsM phylogeny was statistically significant, approximately unbiased (AU) test implemented in CONSEL package^88^ was conducted as reported in a previous study^27^. Briefly, 48 alternative trees where all eukaryotic species were forced into monophyly were generated by prune and re-grafting algorithm, keeping those closest to the original maximum likelihood tree in terms of Robinson and Folds distance (as computed by treedist program in PHYLIP^89^ package). PHYML^90^ was used to optimize parameters and calculate per-site likelihoods (using option -print_site_lnl) by providing the alternative trees as user tree. Programs makemt and catpv implemented in CONSEL package were run to calculate the P value for each alternative topology using the AU test.

### Molecular evolutionary analysis

To examine whether differences in the evolutionary rates among the lineages are significant, we performed a likelihood ratio test (LRT) between homogeneous rate model and heterogeneous rate model using PAML^91^ package. Homogeneous rate assumes a single evolutionary rate (homogeneous rate) for all compared lineages. Heterogeneous rate model assigned each compared lineage with its independent evolutionary rate (heterogeneous rate). The likelihood of two models was calculated with the codeml program in PAML and compared using the LRT. Relative rate test implemented in RRTree^92^ was conducted to confirm whether all compared lineages have a homogeneous rate of evolution.

### Note added in proof

While this manuscript was under review, a new report was published which assessed the evolutionary origin of AS3MT^93^, the animal version of arsenite methyltransferase. In that study, horizontal gene transfer of AS3MT between animal and bacteria as well as between fungi and bacteria was reported. Their conclusions support our concept that recurrent horizontal transfer of *arsM* genes facilitated adaptation of life to arsenic.

## Electronic supplementary material

supporting information
