# Supplementary material for: Recurrent horizontal transfer of arsenite methyltransferase genes facilitated adaptation of life to arsenic
Source: Sci Rep. 2017 Aug 10;7:7741. doi: 10.1038/s41598-017-08313-2 (PMC5552862; doi:10.1038/s41598-017-08313-2)
Supplement: Supplementary file 1 — supporting information [file 41598_2017_8313_MOESM1_ESM.doc]

**Recurrent horizontal transfer of arsenite methyltransferase genes facilitated adaptation of life to arsenic**

Song-Can Chen^a,e^, Guo-Xin Sun^a^, Barry P. Rosen^b^, Si-Yu Zhang^c^, Ye Deng^a^, Bo-Kai Zhu^f^, Christopher Rensing^d^, Yong-Guan Zhu^a,d^*

^a^ State Key Lab of Urban and Regional Ecology, Research Center for Eco-Environmental Sciences, Chinese Academy of Sciences, Beijing 100085, China.

^b^ Department of Cellular Biology and Pharmacology, Herbert Wertheim College of Medicine, Florida International University, Miami, Florida 33199, United States

^c^ School of Civil and Environmental Engineering, Georgia Institute of Technology, 311 Forest Dr., Atlanta, GA 30332-0512, United States

^d^ Key Lab of Urban Environment and Health, Institute of Urban Environment, Chinese Academy of Sciences, Xiamen 361021, China.

^e^ University of Chinese Academy of Sciences, Beijing 100049, China.

^f^ College of Agriculture and Life Science, Cornell University, Ithaca 14853, United States.

* Corresponding author. E-mail: ygzhu@rcees.ac.cn


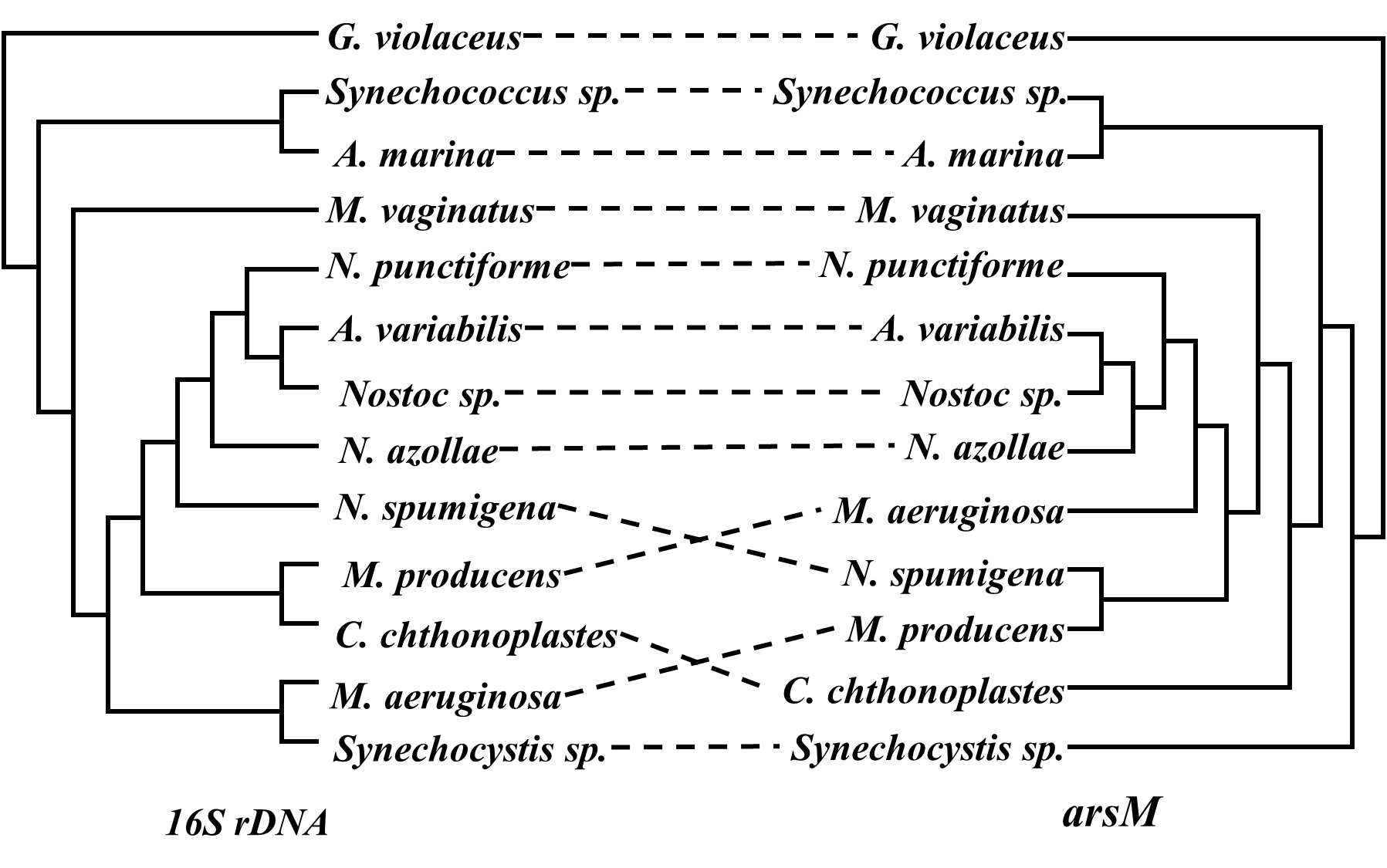


Figure S1. Tanglegram comparing 16S rRNA and arsM genes.


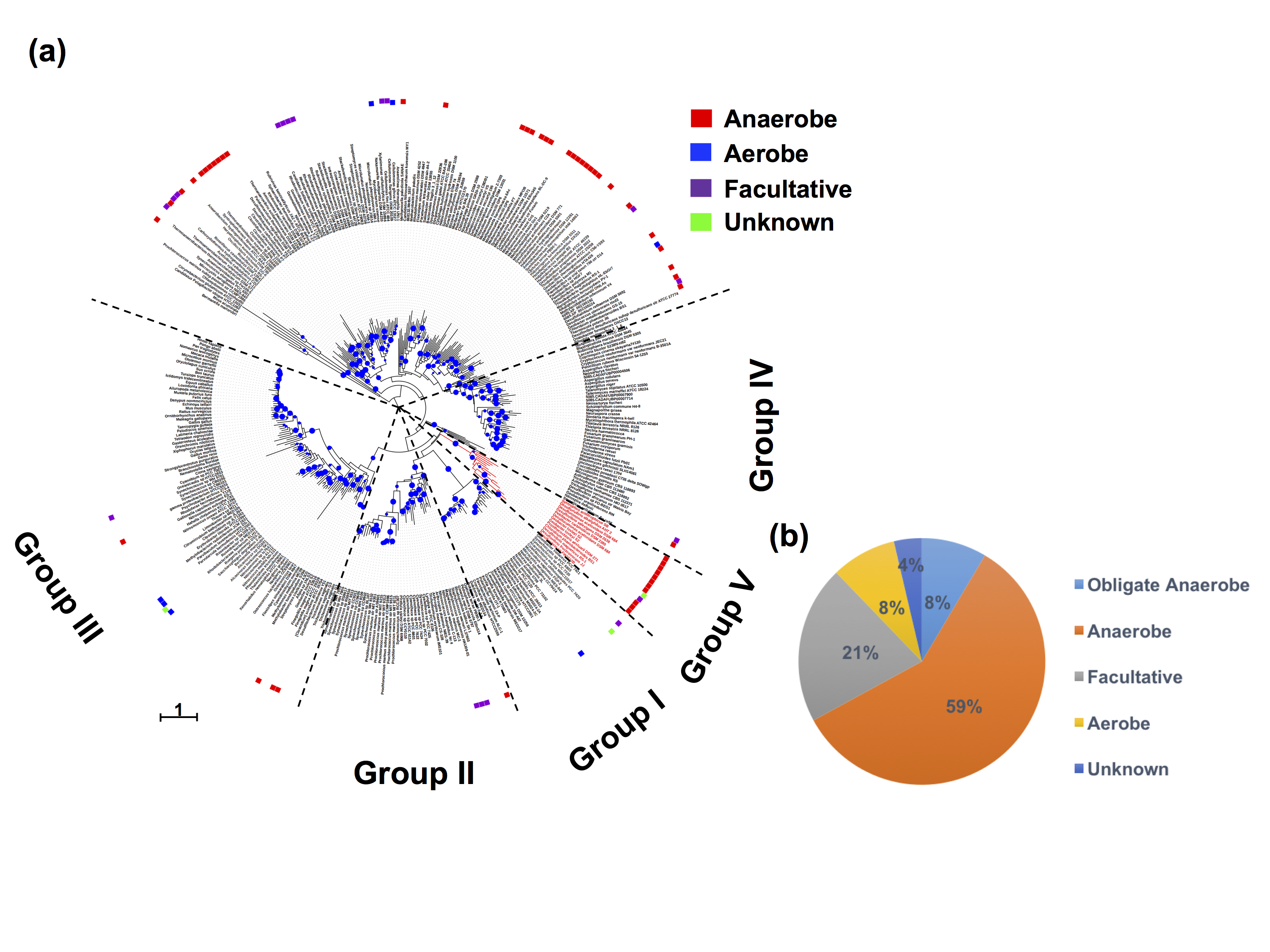


Figure S2. Co-existence analysis of ArsP and ArsM. (a) Mapping of organisms with both ArsP and ArsM back to ArsM phylogeny. The organisms possess both ArsP and ArsM are indicated by squares, whose oxygen requirement (Anaerobe, Facultative, Aerobe) was shown in different colors; (b) Oxygen requirement of organisms with both ArsP and ArsM.


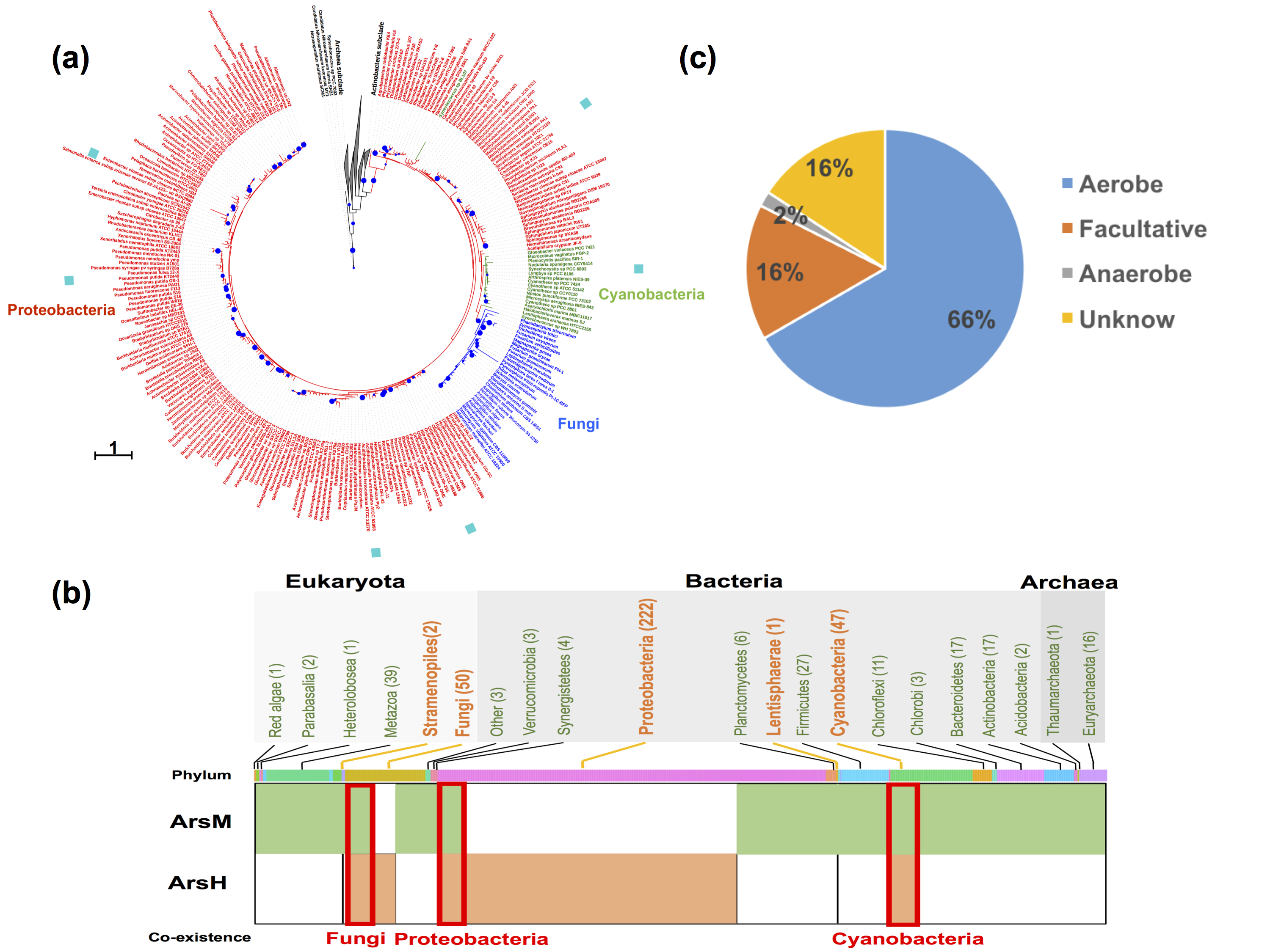


Figure S3. Co-existence analysis of ArsH and ArsM. (a) Phylogeny of ArsH homologs. The phylogenetic tree was constructed using the maximum likelihood program RAxML. The statistical significance of the branch pattern was estimated by conducting 100 bootstrap replications of the original amino acid alignment; bootstraps >50 were shown as blue circle. Proteins with known methylarsenite oxidase (ArsH) function were indicated by blue squares. The monophyletic clade which includes sequences of known ArsH function was shown in color; (b) Taxonomic distribution of ArsH and ArsM. Organisms with co-existence of ArsH and ArsM in genomes were shown within red boxes. (c) Oxygen requirement of organisms with both ArsH and ArsM.


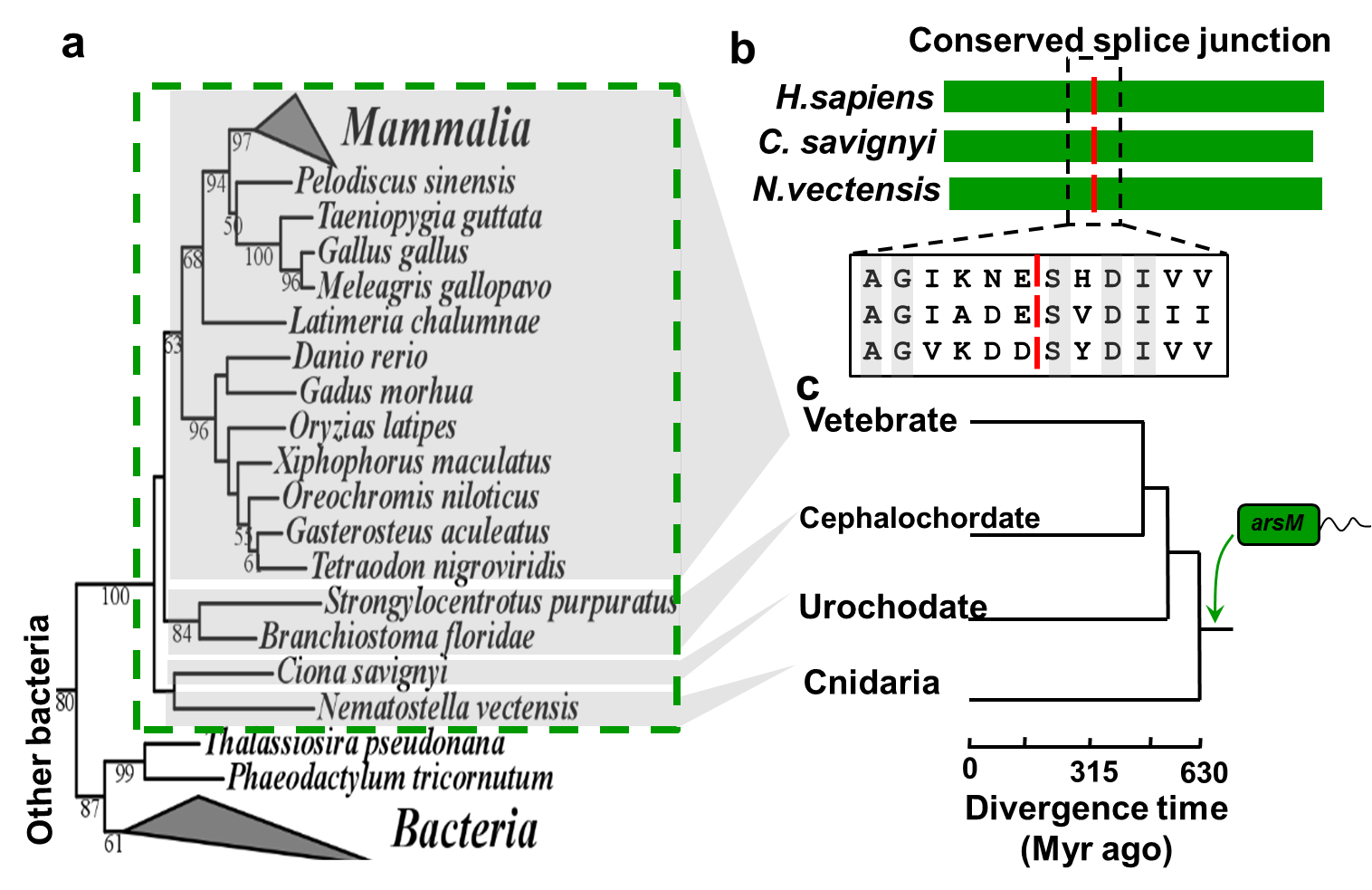


Figure S4. Horizontal gene transfer of *arsM* genes from bacteria to metazoan. a) Maximum likelihood tree of ArsM from metazoan. Maximum likelihood bootstrap support value (>50) are indicated below branches. Dashed lines highlight HGT events; b) Schematic alignment of Vertebrate (*H. sapiens*), Urochodate (*C. savignyi*) and Cnidaria (*N. vectensis*) *arsM* genes with shared (red line) intron position denoted. Aligned residues surrounding the splice site are shown (boxed) with conserved amino acids indicated (grey); c) Metazoan phylogeny with approximate dates of divergence based on concordance with the ArsM tree (1).


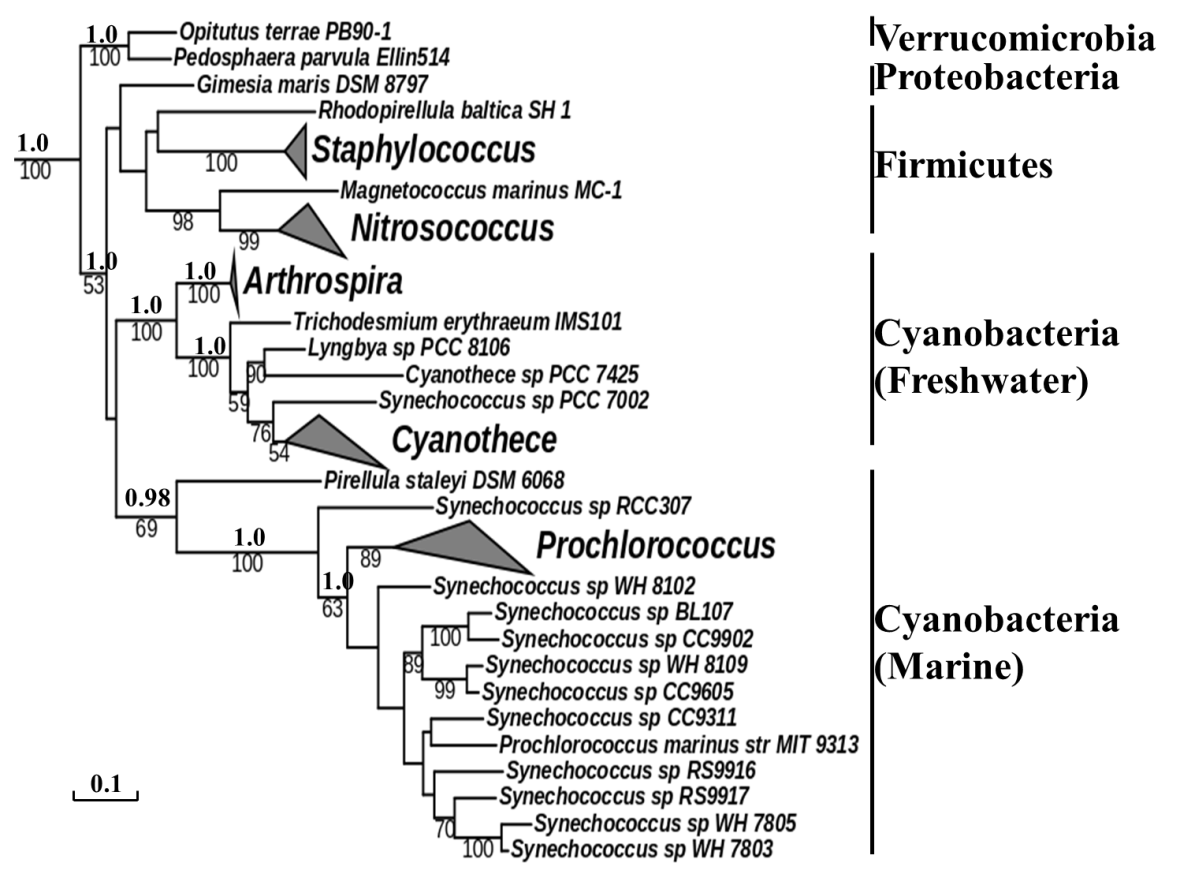


Figure S5. Maximum likelihood tree of ArsM orthologs in group II. Maximum likelihood bootstrap support value (>50) are indicated below branches. Bayesian posterior probability values of critical bipartition appear above the branch for comparison.


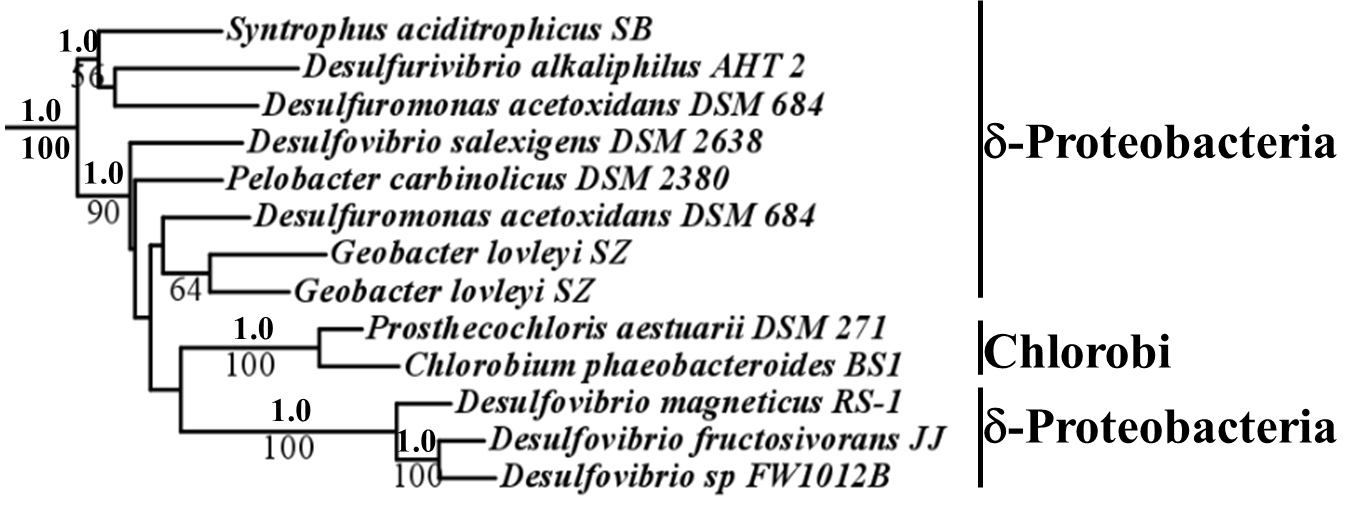


Figure S6. Maximum likelihood tree of ArsM in group V. Maximum likelihood bootstrap support value (>50) are indicated below branches. Bayesian posterior probability values of critical bipartition appear above the branch for comparison.


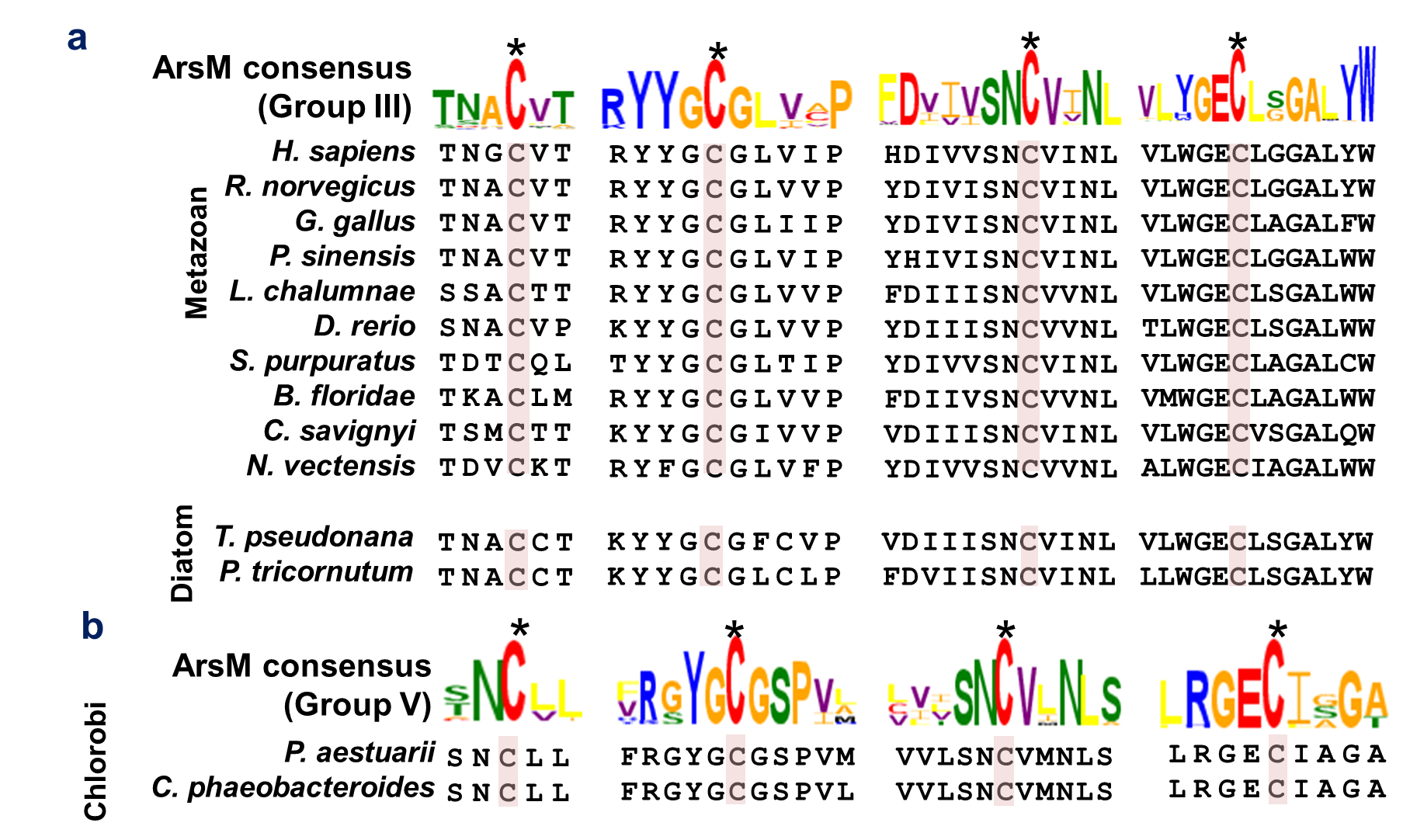


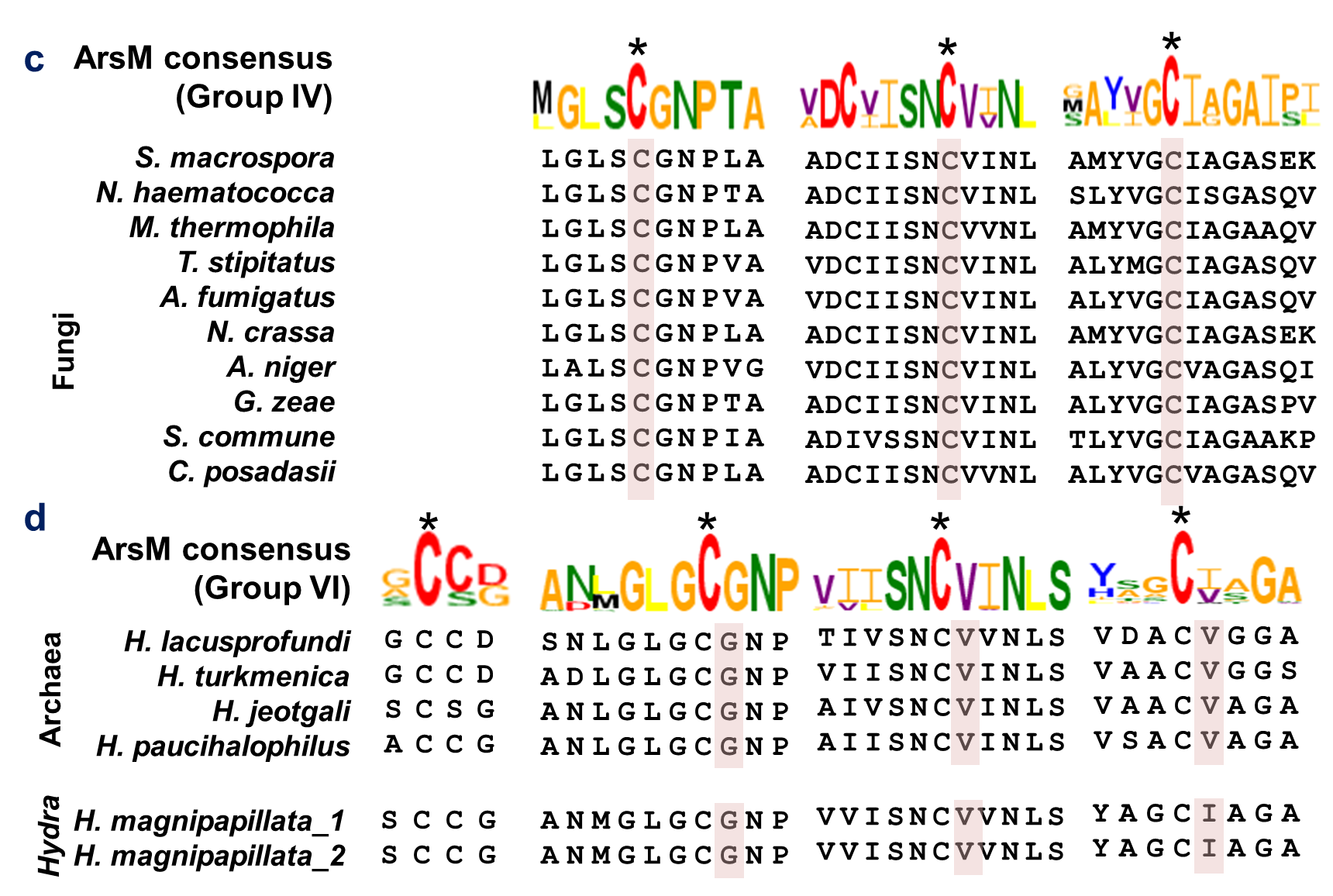


Figure S7. Evidence for retention of important arsenite binding motifs of *arsM* after HGT. **a-d**), alignments for arsenite binding motifs are shown for each group (III-VI). The consensus sequence logo of residues surrounding the cysteine positions of arsenite binding motifs from extant bacterial ArsM enzymes are shown above alignments from each groups. Below are aligned ArsM proteins from HGT hosts in these same regions. Representatives derived from distinct HGT events are separated by a space.

Table S1 Approximate Unbiased (AU) Test between Maximum likelihood tree (unconstrained) against alternative tree(s) which forced all eukaryotic ArsM as monophyletic group (constrained).

| Tree | ln *L* ^a^ | ∆ ln*L* ^b^ | Approximate Unbiased-Test (P value) |
| --- | --- | --- | --- |
| unconstrained tree | -112824.09 | (best) |  |
| alternative tree 1 | -113340.89 | 516.80 | 4E-85* |
| alternative tree 2 | -113342.89 | 518.80 | 1E-39* |
| alternative tree 3 | -113343.69 | 519.60 | 3E-48* |
| alternative tree 4 | -113349.89 | 525.80 | 1E-44* |
| alternative tree 5 | -113350.89 | 526.80 | 7E-8* |
| alternative tree 6 | -113352.89 | 528.80 | 2E-43* |
| alternative tree 7 | -113355.09 | 531.00 | 4E-46* |
| alternative tree 8 | -113421.69 | 597.60 | 3E-8* |
| alternative tree 9 | -113421.69 | 597.60 | 3E-8* |
| alternative tree 10 | -113466.79 | 642.70 | 5E-8* |
| alternative tree 11 | -113466.79 | 642.70 | 5E-8* |
| alternative tree 12 | -113493.49 | 669.40 | 1E-26* |
| alternative tree 13 | -113506.19 | 682.10 | 4E-87* |
| alternative tree 14 | -113509.39 | 685.30 | 8E-87* |
| alternative tree 15 | -113512.19 | 688.10 | 5E-52* |
| alternative tree 16 | -113512.29 | 688.20 | 2E-98* |
| alternative tree 17 | -113512.39 | 688.30 | 1E-67* |
| alternative tree 18 | -113512.99 | 688.90 | 8E-72* |
| alternative tree 19 | -113513.79 | 689.70 | 3E-81* |
| alternative tree 20 | -113514.29 | 690.20 | 6E-99* |
| alternative tree 21 | -113514.39 | 690.30 | 5E-103* |
| alternative tree 22 | -113514.59 | 690.50 | 2E-115* |
| alternative tree 23 | -113515.39 | 691.30 | 1E-55* |
| alternative tree 24 | -113516.49 | 692.40 | 7E-71* |
| alternative tree 25 | -113517.09 | 693.00 | 1E-83* |
| alternative tree 26 | -113517.19 | 693.10 | 2E-121* |
| alternative tree 27 | -113517.19 | 693.10 | 2E-122* |
| alternative tree 28 | -113517.29 | 693.20 | 6E-102* |
| alternative tree 29 | -113517.69 | 693.60 | 4E-100* |
| alternative tree 30 | -113518.69 | 694.60 | 1E-74* |
| alternative tree 31 | -113518.89 | 694.80 | 6E-96* |
| alternative tree 32 | -113520.19 | 696.10 | 1E-174* |
| alternative tree 33 | -113522.49 | 698.40 | 4E-120* |
| alternative tree 34 | -113526.39 | 702.30 | 2E-123* |
| alternative tree 35 | -113526.99 | 702.90 | 1E-9* |
| alternative tree 36 | -113526.99 | 702.90 | 2E-7* |
| alternative tree 37 | -113528.29 | 704.20 | 2E-67* |
| alternative tree 38 | -113529.09 | 705.00 | 2E-78* |
| alternative tree 39 | -113531.19 | 707.10 | 3E-39* |
| alternative tree 40 | -113531.79 | 707.70 | 8E-112* |
| alternative tree 41 | -113532.39 | 708.30 | 0.00000002* |
| alternative tree 42 | -113533.19 | 709.10 | 0.0000002* |
| alternative tree 43 | -113533.69 | 709.60 | 3E-86* |
| alternative tree 44 | -113535.89 | 711.80 | 0.0000002* |
| alternative tree 45 | -113536.29 | 712.20 | 0.00000003* |
| alternative tree 46 | -113536.59 | 712.50 | 0.00000004* |
| alternative tree 47 | -113544.89 | 720.80 | 8E-64* |
| alternative tree 48 | -113545.29 | 721.20 | 4E-63* |

^a^: log likelihood of each phylogenetic tree

^b^: difference of log likelihood between original tree and alternative tree(s).

*: P<0.01

Table S2 Gene structure for eukaryotic *arsM*.

| **Species** | **Protein id^a^** | **Gene id^b^** | **Intron** | **Neighborhood gene id** |
| --- | --- | --- | --- | --- |
| ***Metazoa (Group III)*** |  |  |  |  |
| Branchiostoma floridae | 7739.JGI124020 | 7235107 (NCBI) | 10 Exons |  |
| Oryzias latipes | 8090.ENSORLP00000016161 | ENSORLT00000016162 | 11 Exons |  |
| Xiphophorus maculatus | 8083.ENSXMAP00000000059 | ENSXMAT00000000059 | 11 Exons |  |
| Oreochromis niloticus | 8128.ENSONIP00000023704 | ENSONIT00000023725 | 11 Exons |  |
| Gasterosteus aculeatus | 69293.ENSGACP00000024144 | ENSGACT00000024192 | 11 Exons |  |
| Tetraodon nigroviridis | 99883.ENSTNIP00000000777 | ENSTNIT00000002917 | 12 Exons |  |
| Gadus morhua | 8049.ENSGMOP00000016748 | ENSGMOT00000017167 | 11 Exons |  |
| Danio rerio | 7955.ENSDARP00000057878 | ENSDART00000057879 | 11 Exons |  |
| Latimeria chalumnae | 7897.ENSLACP00000006737 | ENSLACT00000006791 | 12 Exons |  |
| Ornithorhynchus anatinus | 9258.ENSOANP00000002637 | ENSOANT00000002638 | 9 Exons |  |
| Loxodonta africana | 9785.ENSLAFP00000010070 | ENSLAFT00000012046 | 11 Exons |  |
| Echinops telfairi | 9371.ENSETEP00000006260 | ENSETET00000007706 | 11 Exons |  |
| Rattus norvegicus | 10116.ENSRNOP00000027223 | ENSRNOT00000027223 | 11 Exons |  |
| Mus musculus | 10090.ENSMUSP00000003655 | ENSMUST00000003655 | 11 Exons |  |
| Felis catus | 9685.ENSFCAP00000020766 | ENSFCAT00000028372 | 9 Exons |  |
| Mustela putorius furo | 9669.ENSMPUP00000016746 | ENSMPUT00000016995 | 10 Exons |  |
| Ailuropoda melanoleuca | 9646.ENSAMEP00000018882 | ENSAMET00000019637 | 10 Exons |  |
| Oryctolagus cuniculus | 9986.ENSOCUP00000000542 | ENSOCUT00000000625 | 13 Exons |  |
| Macaca mulatta | 9544.ENSMMUP00000010070 | ENSMMUT00000010734 | 10 Exons |  |
| Pongo abelii | 9601.ENSPPYP00000003042 | ENSPPYT00000003146 | 13 Exons |  |
| Homo sapiens | 9606.ENSP00000358896 | ENST00000369880 | 11 Exons |  |
| Pan troglodytes | 9598.ENSPTRP00000006699 | ENSPTRT00000007262 | 11 Exons |  |
| Nomascus leucogenys | 61853.ENSNLEP00000018465 | ENSNLET00000019390 | 9 Exons |  |
| Otolemur garnettii | 30611.ENSOGAP00000003876 | ENSOGAT00000004344 | 10 Exons |  |
| Microcebus murinus | 30608.ENSMICP00000011185 | ENSMICT00000012285 | 11 Exons |  |
| Ictidomys tridecemlineatus | 43179.ENSSTOP00000012979 | ENSSTOT00000014488 | 14 Exons |  |
| Tursiops truncatus | 9739.ENSTTRP00000011978 | ENSTTRT00000012624 | 14 Exons |  |
| Bos taurus | 9913.ENSBTAP00000047294 | ENSBTAT00000050619 | 11 Exons |  |
| Sus scrofa | 9823.ENSSSCP00000011284 | ENSSSCT00000011586 | 11 Exons |  |
| Equus caballus | 9796.ENSECAP00000016550 | ENSECAT00000020180 | 11 Exons |  |
| Dasypus novemcinctus | 9361.ENSDNOP00000004455 | ENSDNOT00000005754 | 9 Exons |  |
| Taeniopygia guttata | 59729.ENSTGUP00000010561 | ENSTGUT00000010672 | 8 Exons |  |
| Gallus gallus | 9031.ENSGALP00000013184 | ENSGALT00000013199 | 11 Exons |  |
| Meleagris gallopavo | 9103.ENSMGAP00000010803 | ENSMGAT00000011668 | 10 Exons |  |
| Pelodiscus sinensis | 13735.ENSPSIP00000004362 | ENSPSIT00000004385 | 9 Exons |  |
| Ciona savignyi | 51511.ENSCSAVP00000001593 | ENSCSAVT00000001615 | 9 Exons |  |
| Strongylocentrotus purpuratus | 7668.SPU_001668-tr | SPU_001668 | 8 Exons |  |
| Nematostella vectensis | 45351.NEMVEDRAFT_v1g98842 | NEMVEDRAFT_v1g98842 | 9 Exons |  |
| ***Fungi (Group IV)*** |  |  |  |  |
| Aspergillus clavatus | 5057.CADACLAP00005923 | CADACLAT00006060 | 5 Exons |  |
| Neosartorya fischeri | 36630.CADNFIAP00000204 | CADNFIAT00000213 | 5 Exons |  |
| Aspergillus fumigatus | 5085.CADAFUBP00004506 | CADAFUBT00004584 | 2 Exons |  |
| Emericella nidulans | 162425.CADANIAP00005808 | CADANIAT00005808 | 5 Exons |  |
| Aspergillus fumigatus | 5085.CADAFUBP00007900 | CADAFUBT00008040 | 4 Exons |  |
| Aspergillus fumigatus | 5085.CADAFUBP00007714 | CADAFUBT00007851 | 4 Exons |  |
| Neosartorya fischeri | 36630.CADNFIAP00008848 | CADNFIAT00009062 | 5 Exons |  |
| Aspergillus terreus | 33178.CADATEAP00010154 | CADATEAT00010154 | 3 Exons |  |
| Aspergillus niger | 5061.CADANGAP00004769 | An05g01160 | 3 Exons |  |
| Penicillium chrysogenum Wisconsin 54 1255 | 500485.XP_002559277.1 | 8310354 (NCBI) | 4 Exons |  |
| Penicillium marneffei ATCC 18224 | 441960.XP_002153549.1 | 7030909 (NCBI) | 4 Exons |  |
| Talaromyces stipitatus ATCC 10500 | 441959.XP_002487876.1 | 8102897 (NCBI) | 5 Exons |  |
| Ajellomyces dermatitidis SLH14081 | 559298.XP_002628829.1 | 8508120 (NCBI) | 5 Exons |  |
| Ajellomyces capsulatus NAm1 | 339724.XP_001543203.1 | 5450067 (NCBI) | 5 Exons |  |
| Arthroderma otae CBS 113480 | 554155.XP_002843162.1 | 9227361 (NCBI) | 5 Exons |  |
| Arthroderma otae CBS 113480 | 554155.XP_002843162.1 | 9227361 (NCBI) | 5 Exons |  |
| Arthroderma gypseum CBS 118893 | 535722.XP_003173062.1 | 10028339 (NCBI) | 4 Exons |  |
| Arthroderma benhamiae CBS 112371 | 663331.XP_003014635.1 | 9526998 (NCBI) | 5 Exons |  |
| Trichophyton rubrum CBS 118892 | 559305.XP_003237817.1 | 10379200 (NCBI) | 5 Exons |  |
| Trichophyton verrucosum HKI 0517 | 663202.XP_003019045.1 | 9582438 (NCBI) | 5 Exons |  |
| Uncinocarpus reesii 1704 | 336963.XP_002542608.1 | 8441320 (NCBI) | 5 Exons |  |
| Coccidioides immitis RS | 246410.XP_001240610.1 | 4560650 (NCBI) | 9 Exons |  |
| Coccidioides posadasii C735 delta SOWgp | 222929.XP_003067903.1 | 9693386 (NCBI) | 4 Exons |  |
| Paracoccidioides sp. lutzii Pb01 | 502779.XP_002796696.1 | 9099999 (NCBI) | 4 Exons |  |
| Hypocrea jecorina | 51453.JGI53428 | 18485536 (NCBI) | 4 Exons |  |
| Hypocrea virens | 29875.EHK18292 | TRIVIDRAFT_194105 | 5 Exons |  |
| Fusarium oxysporum | 5507.FOXG_13346P0 | FOXG_13346T0 | 4 Exons |  |
| Gibberella zeae | 5518.FGSG_04845P0 | FG04845.1 | 5 Exons |  |
| Gibberella zeae PH 1 | 229533.XP_385021.1 | 2788115 | 5 Exons |  |
| Necteria haematococca | 140110.NechaP57802 | NechaT57802 | 5 Exons |  |
| Gaeumannomyces graminis | 29850.GGTG_09078T0 | GGTG_09078T0 | 5 Exons |  |
| Magnaporthe grisea | 148305.A4RGS3 | 640284968 (JGI) | 2 Exons |  |
| Myceliophthora thermophila ATCC 42464 | 573729.XP_003663971.1 | 11513446 (NCBI) | 3 Exons |  |
| Thielavia terrestris NRRL 8126 | 578455.XP_003650075.1 | 11516603 (NCBI) | 5 Exons |  |
| Thielavia terrestris NRRL 8126 | 578455.XP_003657483.1 | 11523052 (NCBI) | 5 Exons |  |
| Neurospora crassa | 5141.EFNCRP00000005499 | NCU05616 | 2 Exons |  |
| Sordaria macrospora k hell | 771870.XP_003353016.1 | 10810671 (NCBI) | 2 Exons |  |
| Coprinopsis cinerea okayama7_130 | 240176.XP_001832959.2 | 6009450 (NCBI) | 9 Exons |  |
| Schizophyllum commune H4 8 | 578458.XP_003037640.1 | 9594997 (NCBI) | 4 Exons |  |
| Laccaria bicolor S238N H82 | 486041.XP_001888258.1 | 6083936 (NCBI) | 6 Exons |  |
| Cryptococcus neoformans var. neoformans JEC21 | 214684.XP_567425.1 | 3254203 (NCBI) | 4 Exons |  |
| Cryptococcus neoformans var. neoformans B 3501A | 283643.XP_773003.1 | 4938622 (NCBI) | 4 Exons |  |
| ***Metazoa (Group VI)*** |  |  |  |  |
| Hydra magnipapillata | 6085.XP_002165192 | 100203411 (NCBI) | No | 100199814 (2 Exon) |
| Hydra magnipapillata | 6085.XP_002165224 | 105843548 (NCBI) | 2 Exons |  |

^a^: Protein id from EggNOG database

^b^: Gene id from Ensembl database or Entrezgene database.

Table S3 Likelihood value of homogeneous rate model and heterogeneous rate model estimated with PAML

|  |  |  | LRT | |
| --- | --- | --- | --- | --- |
| Model | np | ln *L* | X^2^=∆ ln *L* | *P* value |
| Homogeneous rate model^a^ | 343 | -117482.65 |  |  |
| Heterogeneous rate model^b^ | 346 | -117481.95 | 1.395678 | 0.7065 (df=3) |

np: number of parameters in the model.

ln *L*: log likelihood of the each assumed model.

^a^: In homogeneous rate model, same evolutionary rate was assigned to the compared lineages.

^b^: In heterogeneous rate model, independent evolution rate was assumed among compared lineages.

Table S4 Relative rate test among three eukaryotic lineages using RRTree

|  | Average distrance (dK) | | Standard deviation (sd) | dK/ds | P value |
| --- | --- | --- | --- | --- | --- |
| Metazoa^a^ vs. Fungi^b^ | -0.10 | | 0.15 | -0.68 | 0.40 |
| Metazoa vs. Metazoa 1^c^ | | -0.15 | 0.18 | -0.83 | 0.40 |
| Fungi vs. Metazoa | -0.05 | | 0.16 | -0.29 | 0.77 |

^a^: Metazoan ArsM in Group III

^b^: Fungal ArsM in Group IV

^c^: Metazoan ArsM in Group VI

Table S5 Evolutionary analysis of *arsM* gene groups.

|  | **Gene** | **Species** | **Numer of species** | **dN/dS** | **Codons evolving under purifying selection** | |  |
| --- | --- | --- | --- | --- | --- | --- | --- |
| Eukaryotic *arsM* | Group III | Metazoan | 33 | 0.27 | | 44% (143 of 324) | |
|  | Group III | Diatom | 3 | 0.16 | | 1% (5 of 412) | |
|  | Group IV | Fungi | 38 | 0.16 | | 76% (152 of 201) | |
| Archaea *arsM* | Group VI | Halobacteria | 4 | 0.19 | | 2% (5 of 234) | |
| Bacterial *arsM* | Group II | Cyanobacteria | 16 | 0.15 | | 52% (132 of 254) | |
|  | Group III | Proteobacteria | 31 | 0.18 | | 73% (238 of 326) | |
|  | Group IV | Planctomycetes | 3 | 0.17 | | 0.3% (1 of 265) | |
|  | Group V | Anaerobes | 11 | 0.26 | | 31% (271 of 888) | |
|  | Group VI | Bacteria | 18 | 0.17 | | 61% (139 of 229) | |

Summary of results from maximum likelihood tests of aligned *arsM* sequences from the indicated species using SLAC in the HyPhy software package. The overall gene dN/dS ratio (ratio of nonsynonymous changes to synonymous changes) is shown, indicating an overall signature of purifying selection. Individual codons with a statistically significant signature of purifying selection (*P* < 0.05) were also calculated and are expressed as a percentage of the total number of codons used in the analysis. In the same analysis, no codons were found with a statistically significant signature of positive selection.

Reference

1. Kuraku, S., Meyer, A., & Kuratani, S. Timing of genome duplications relative to the origin of the vertebrates: did cyclostomes diverge before or after? *Mol Biol Evol* **26**, 47-59 (2009).
